# Inhalation arsine poisoning: clinical features and treatment experience of 12 cases from a single incident

**DOI:** 10.1186/s40360-026-01199-9

**Published:** 2026-08-19

**Authors:** Wei Hu, Juan Yang, Xiaoxia Lu, Lili Bai, Xiaobo Peng, Mingfei Peng, Xigang Zhang, Yanqing Liu

**Affiliations:** 1 Department of Emergency, The Fifth Medical Center of Chinese PLA General Hospital, Beijing, 100039 China; 2 Senior Department of Infectious Diseases, The Fifth Medical Center of Chinese PLA General Hospital, National Clinical Research Center for Infectious Diseases, Beijing, 100039 China; 3 Poisoning Treatment Department, Senior Department of Hematology, The Fifth Medical Center of PLA General Hospital, Beijing, 100071 China

**Keywords:** Arsine, Arsenic, Poisoning, Hemolysis, Blood purification, Methylprednisolone, Diagnosis

## Abstract

**Background:**

Arsine poisoning is a rare but life-threatening condition characterized by severe hemolysis. Clinical data on its management remain limited. This study describes the clinical features, diagnostic approach, and treatment experience of 12 patients from a single arsine poisoning incident.

**Methods:**

Clinical data from the 12 patients were retrospectively collected. Presenting symptoms, diagnostic findings, and treatment strategies were summarized.

**Results:**

Ten of the 12 patients survived. Arsenic was detected in cleaning wastewater and in the red blood cells of four patients, and, together with clinical hemolysis, supported the diagnosis of arsine poisoning. Common symptoms included headache, nausea, vomiting, abdominal pain, dizziness, chest tightness, and hematuria; headache, nausea, and vomiting frequently co-occurred. All patients received high-dose methylprednisolone, vitamin C + B6, and chelation therapy. The two non-survivors additionally received mechanical ventilation, therapeutic plasma exchange, dialysis, methylene blue, and transfusions; they died on day 3 from progressive cardiovascular failure secondary to acute hemolysis. Five other patients underwent therapeutic plasma exchange and dialysis and ultimately survived.

**Conclusions:**

This case series highlights the clinical presentation, diagnostic workflow, and management of arsine poisoning in a well-characterized patient cluster. Early recognition and timely intervention may facilitate clinical management. The findings are descriptive and limited by the retrospective design and small sample size.

## Introduction

Although rare, arsine (arsenic hydride, AsH₃) poisoning has been documented in industrial and environmental settings [1, 2]. Arsine poisoning can lead to multi-organ failure and can be lethal [3]. Diagnosing arsine toxicity requires a high index of suspicion and a thorough exposure history [4]. Current management strategies primarily focus on supportive care, blood purification, and, in some cases, chelation therapy, although evidence for these approaches remains limited [5, 6]. The lack of standardized treatment protocols and the complexity of its clinical manifestations often complicate timely diagnosis and intervention. For instance, symptoms such as hematuria, jaundice, and renal failure may mimic other conditions, leading to delays in appropriate treatment. In this study, we present the clinical characteristics, diagnostic workflow, and treatment experience of an arsine poisoning incident that involved 12 patients.

## Methods

This retrospective case series included 12 patients admitted to the Chemical Injury and Poisoning Treatment Department of the Fifth Medical Center of the PLA General Hospital following a single arsine poisoning incident in 2017. The study protocol was approved by the Institutional Ethics Review Committee of the Fifth Medical Center of the PLA General Hospital (Approval No. KY-2025-11-205-1). All procedures were conducted in accordance with the Declaration of Helsinki. The 12 patients involved in this incident were included. No patients were excluded.

Clinical data were extracted retrospectively from electronic medical records using a standardized form. Data collected included patient demographics (age, gender, occupation and comorbidities), exposure history, presenting symptoms, laboratory parameters, treatments administered, and clinical outcomes. The Sequential Organ Failure Assessment (SOFA) score was calculated daily during hospitalization to monitor organ dysfunction. Hemolysis was defined clinically by the presence of hemoglobinuria and/or a rapid decline in hemoglobin (> 2 g/dL within 24 h) without overt bleeding, supported by elevated serum lactate dehydrogenase (LDH) and indirect bilirubin levels.

The diagnosis of arsine poisoning was supported by environmental and biological monitoring. On-site air sampling was performed using a military-grade chemical agent detector (ChemPro 100) and a portable gas detector (Honeywell MultiRAE PGM-6208VOC), employing ion mobility spectrometry and photoionization detection, respectively. Arsenic concentrations in cleaning wastewater and in red blood cells (RBCs) were measured using inductively coupled plasma mass spectrometry. RBC arsenic levels were quantified in four severe patients to confirm systemic arsenic exposure, while the other eight patients did not undergo testing.

All patients received individualized treatment. Anti-inflammatory therapy consisted of high-dose methylprednisolone (500–1000 mg/day). Antioxidant therapy included high-dose vitamin C (10 g/day), vitamin B6 (1000–1200 mg/day), and glutathione supplementation (3.6 g/day). Chelation therapy with sodium dimercaptopropanesulfonate (DMPS) was administered. Blood purification techniques, including therapeutic plasma exchange (TPE) and dialysis, were performed to remove arsenic-hemoglobin complexes and other toxins. When methemoglobinemia was suspected, methylene blue was administered; methemoglobin levels were measured spectrophotometrically from arterial blood samples, with a threshold of 1.5% for intervention. Supportive measures included tracheal intubation with mechanical ventilation and transfusions of red blood cells, platelets, and/or plasma as clinically indicated. Key laboratory parameters, including complete blood count, renal and liver function tests, and coagulation profiles, were monitored serially.

## Results

On September 25, 2017, twelve workers in a 200 m² workshop without ventilation were exposed to irritated gas while cleaning aluminum electrode plates. The cleaning process involved immersing plates coated with industrial oil in a solution of sodium hydroxide (50 kg) and water. During the procedure, bubbles formed and a strong, unpleasant odor was noted. Workers wore rubber gloves, protective shoes, and standard masks, minimizing the risk of dermal exposure. Two workers removed plates from the cleaning solution, eight rinsed them with high-pressure water guns from a distance of approximately one meter, and two conducted patrol inspections.

**Fig. 1:**
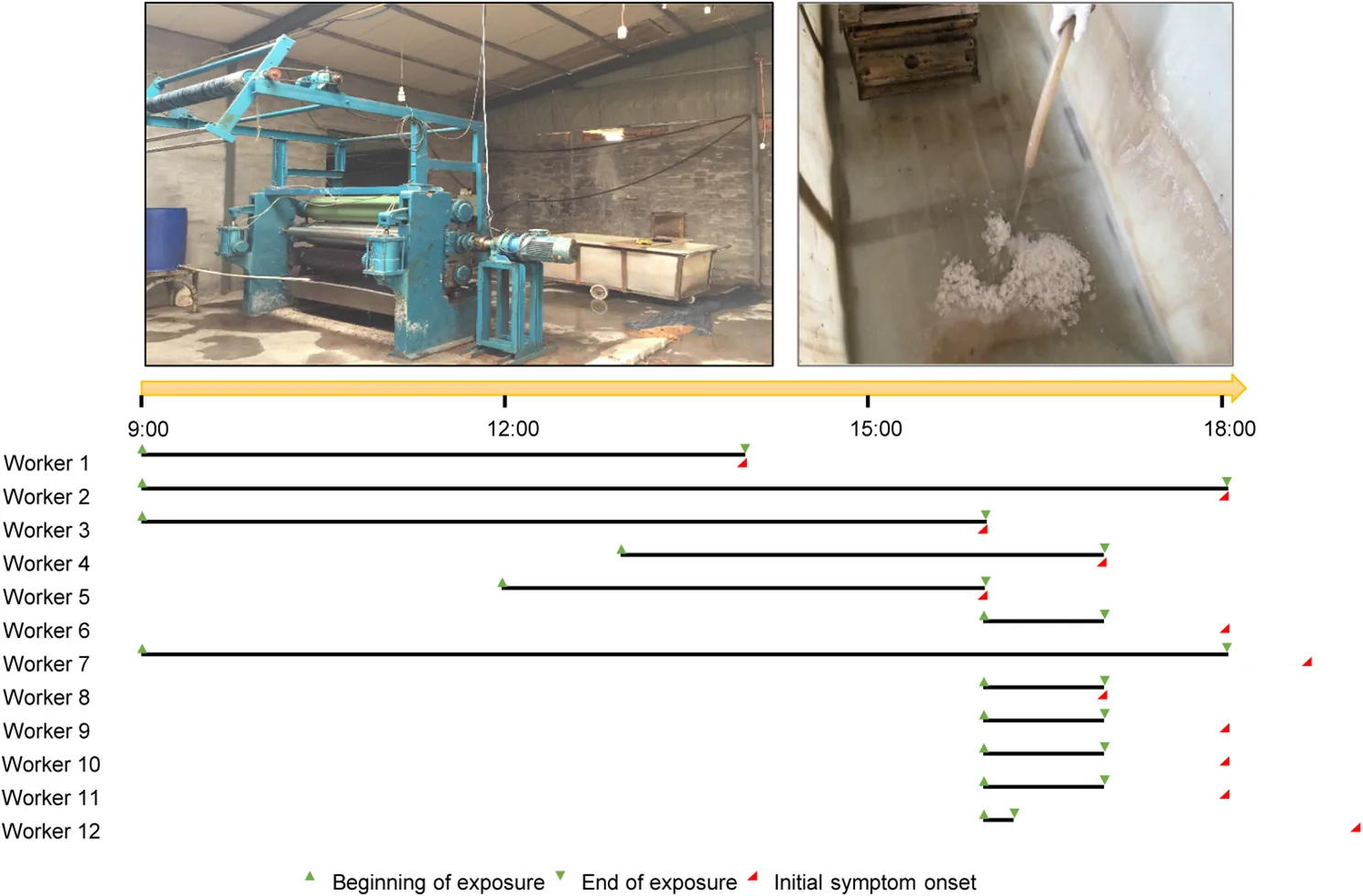
Timeline of inhalation arsine poisoning. Upward green triangles indicate the start of exposure, downward green triangles indicate the end of exposure, and red triangles indicate the time of symptom onset

Figure 1 and Table 1 present the detailed clinical characteristics and exposure history of all 12 patients. The most common presenting symptoms were headache (7 patients), nausea (6 patients), vomiting (5 patients), and abdominal pain (4 patients). Additional symptoms included dizziness (3 patients), hematuria (3 patients), chest tightness (2 patients), fever (1 patient), abdominal distension (1 patient), fatigue (1 patient), and profuse sweating (1 patient). Headache, nausea, and vomiting frequently co-occurred, as did abdominal pain with other gastrointestinal symptoms. Hematuria was noted in three patients on admission, suggesting early renal involvement, while chest tightness and profuse sweating were observed in a few cases, possibly reflecting rapid hemolysis.

**Table 1:** Demographic and clinical characteristics of patients with inhalation arsine poisoning

No.	Age	Gender	Occupation	Comorbidities	Exposure duration (h)	Poisoning to symptom onset (h)	Poisoning to treatment (h)	Initial symptoms	SOFA score
1	59	Male	Salvage	None	5	5	3	Profuse sweating, and chest tightness	11
2	54	Male	Salvage	Grade 3 hypertension (high risk)	9	9	0.5	Dizziness, headache, nausea, vomiting, abdominal pain, and hematuria	10
3	31	Male	Washing	None	7	7	4	Nausea, and vomiting	8
4	27	Male	Washing	None	4	4	0.5	Dizziness, and abdominal pain	7
5	30	Male	Washing	None	4	4	4	Headache, nausea, vomiting	4
6	65	Male	Washing	None	1	2	16	Fever, abdominal distension, and hematuria	8
7	51	Male	Inspection	None	9	10.5	5.5	Vomiting, abdominal pain, and fatigue	3
8	20	Male	Washing	None	1	1	7	Nausea	5
9	50	Male	Washing	HIV infection	1	2	1	Headache	3
10	53	Male	Washing	None	1	2	1	Headache, and nausea	3
11	61	Male	Washing	None	1	2	1	Headache, chest tightness, and hematuria	3
12	36	Male	Inspection	None	0.25	6	8	Headache	2

SOFA, Sequential Organ Failure Assessment

On-site gas sampling detected carbon monoxide at 81 ppm and hydrogen cyanide at 26 ppm. However, carboxyhemoglobin levels were below 4% in all patients, ruling out significant carbon monoxide poisoning. Methemoglobin levels were uniformly below 10%, which, together with the absence of cyanide-typical methemoglobinemia, supported the exclusion of hydrogen cyanide as the primary cause. Cleaning wastewater contained arsenic at 612 mg/L. Arsenic concentrations in RBCs were measured in four severely ill patients, with values of 5.43, 9.49, 9.70, and 10.02 µg/g, respectively. Although arsine itself was not detectable by the on-site detectors, the combination of environmental arsenic detection, clinical hemolysis, and elevated RBC arsenic levels supported the clinical diagnosis of arsine poisoning.

Among the 12 patients, two died (Patients 1 and 2) and ten survived (Table 1). The two non-survivors had SOFA scores of 11 and 10 at admission. Of the survivors, three (Patients 3, 4, and 6) had admission SOFA scores ≥ 7. One additional survivor (Patient 5) had an initial SOFA score of 4 but subsequently deteriorated to a score of 8. Admission laboratory findings are presented in Table 2. Severe cases generally showed marked hematological abnormalities. Patients 1–4 had white blood cell (WBC) counts > 20 × 10⁹/L and neutrophil counts > 17 × 10⁹/L. They also exhibited lower RBC counts, hemoglobin levels, hematocrit, and platelet count. Patient 4, who was hospitalized for 64 days, had an RBC count of 2.67 × 10¹²/L, hemoglobin of 80 g/L, hematocrit of 0.258, and platelets of 199 × 10⁹/L. Patient 5, despite an initial SOFA score of only 4, already displayed elevated WBC and neutrophil counts. In contrast, Patient 6 presented with low hemoglobin and platelet levels despite a normal WBC count, illustrating heterogeneity in laboratory patterns.

**Table 2:** Laboratory findings of patients with inhalation arsine poisoning at admission

No.	WBC count (10^9^/L)	Neutrophil count (10^9^/L)	Neutrophil percentage (%)	RBC count (10^9^/L)	Hb (g/L)	Hematocrit (%)	PLT count (10^9^/L)	RBC arsenic concentrations (µg/g)
1	25.76	23.18	0.9	2.96	93	0.283	40	9.7
2	20.84	17.77	0.853	2.87	82	0.257	106	9.49
3	24.8	19.63	0.792	3.49	84	0.326	257	10.02
4	24.57	20.99	0.854	2.67	80	0.258	199	5.43
5	21.95	18.47	0.842	3.36	107	0.343	255	Not measured
6	9.22	7.63	0.828	2.33	82	0.221	69	Not measured
7	9.27	7.94	0.856	3.62	125	0.401	134	Not measured
8	17.15	14.77	0.861	2.92	92	0.278	243	Not measured
9	5	3.6	0.731	2.8	91	0.279	121	Not measured
10	9.5	8	0.841	3.84	115	0.358	182	Not measured
11	9.9	7.5	0.754	2.98	94	0.285	156	Not measured
12	13	11.9	0.912	4.18	127	0.385	250	Not measured

WBC, white blood cell; RBC, red blood cell; Hb, hemoglobin; PLT, platelet

All patients received high-dose methylprednisolone, vitamin C + B6, and DMPS. Both non-surviving patients (Patients 1 and 2) received intensive care and additional tracheal intubation with mechanical ventilation, TPE, dialysis, methylene blue, and transfusions of red blood cells, platelets, and plasma (Table 3). They died on the third hospital day due to progressive cardiovascular failure secondary to acute hemolysis. The other four patients (Patients 3, 4, 5, and 6) had severe disease or disease deterioration and thus underwent TPE and dialysis along with other supportive measures (Table 3). Another patient (Patient 8) also received TPE and dialysis because of a critically low hemoglobin level (60 g/L) despite an otherwise stable clinical status. The remaining five patients had admission SOFA scores below 7, remained clinically stable, and received no additional treatment.

**Table 3:** Major treatment and clinical outcome of patients with inhalation arsine poisoning

No.	Major treatment	Hospital stay (days)	Outcome
1	Dialysis filtration, TPE, MP, MB, VitC + VitB6, transfusion of RBCs, transfusion of PLTs, transfusion of plasma, tracheal intubation with ventilator-assisted breathing	3	Deceased
2	Dialysis filtration, TPE, MP, MB, VitC + VitB6, transfusion of plasma, tracheal intubation with ventilator-assisted breathing	3	Deceased
3	Dialysis filtration, TPE, MP, MB, VitC + VitB6, DMPS, transfusion of RBCs, transfusion of PLTs, transfusion of plasma	71	Survival
4	Dialysis filtration, TPE, MP, MB, VitC + VitB6, combined plasma and red blood cell exchange, DMPS, transfusion of RBCs, transfusion of plasma, transfusion of PLTs	64	Survival
5	Dialysis filtration, TPE, MP, MB, VitC + VitB6, DMPS, transfusion of RBCs, transfusion of plasma	41	Survival
6	Dialysis filtration, TPE, MP, MB, VitC + VitB6, transfusion of RBCs, transfusion of plasma	69	Survival
7	MP, MB, VitC + VitB6, DMPS, transfusion of RBCs	41	Survival
8	Dialysis filtration, TPE, MP, MB, VitC + VitB6, DMPS, transfusion of RBCs, transfusion of plasma	31	Survival
9	MP, VitC + VitB6, DMPS	20	Survival
10	MP, VitC + VitB6, DMPS	20	Survival
11	MP, VitC + VitB6, DMPS	20	Survival
12	MP, VitC + VitB6, DMPS	16	Survival

TPE, therapeutic plasma exchange; DMPS, sodium Dimercaptopropanesulfonate; RBCs, red blood cells; MP, methylprednisolone; MB, methylene blue; PLTs, platelets; VitC, vitamin C; VitB6, vitamin B6

## Discussion

In this study, we summarized the clinical experiences and treatment outcomes of an arsine poisoning incident involving 12 patients. The analysis was motivated by our recent management of new arsine poisoning cases, which highlighted the persistent scarcity of clinical experience in the literature. Our report provides a comprehensive description of the diagnostic workflow, including environmental and biological monitoring, and the clinical management strategies employed, including high-dose corticosteroid therapy combined with blood purification.

Our study has several limitations. First, RBC arsenic levels were measured in only four patients, limiting analysis of the correlation between toxicant exposure and clinical severity. Second, the presence of other gases at the exposure site suggests possible mixed toxic exposure, which may have contributed to the clinical presentation. Third, the absence of a control group and the observational design preclude any definitive conclusions regarding treatment efficacy. Despite these limitations, this case series provides valuable real-world insights into the diagnosis and management of arsine poisoning and contributes to the limited literature [5, 7].

Arsine gas is a highly toxic byproduct that can be generated during industrial processes involving arsenic-containing materials. In this incident, arsine was produced when sodium hydroxide was used to clean aluminum electrode plates. The chemical reaction between aluminum and sodium hydroxide releases atomic hydrogen, which subsequently reacts with arsenic present in the environment to form arsine gas. This incident underscores the critical need for public awareness and preventive measures to avoid such exposures. Following the incident, the combination of environmental arsenic detection, clinical hemolysis, and elevated RBC arsenic levels supported the clinical diagnosis of arsine poisoning and guided further clinical treatment.

Arsine toxicity primarily arises from its interaction with hemoglobin. Upon inhalation, arsine binds to hemoglobin and is oxidized to dihydrogen arsenide and elemental arsenic, resulting in acute intravascular hemolysis. This process leads to the depletion of glutathione stores. While hemoglobin-haptoglobin complex formation is a protective mechanism that prevents renal injury by clearing free hemoglobin, an overwhelming hemolytic crisis can exceed this capacity, leading to hemoglobinuria and direct tubular injury from hemoglobin casts [3]. The distinct toxicity of arsine, compared to other forms of arsenic exposure, is attributed to its capacity to induce rapid and severe hemolysis through oxidative damage to hemoglobin and red cell membranes, and inhibition of red blood cell enzymes like Na+/K+ ATPase [3, 8, 9]. These mechanisms elucidate the severe clinical manifestations observed in this incident. Severe patients displayed progressive hemolysis as well as reduced platelet counts and elevated WBC and neutrophil counts, highlighting the necessity for close monitoring of these parameters in affected individuals.

Acute arsine poisoning is characterized by rapid-onset symptoms, which may include gastrointestinal distress, hematuria, and a bronze or red discoloration of the skin due to hemolysis [5, 10, 11]. Severe cases can develop multi-organ dysfunction, manifesting as acute kidney injury, hepatic dysfunction, and cardiovascular collapse within hours to days [12]. In this study, the most frequently reported symptoms among patients included headache, nausea, vomiting, and abdominal pain. Tragically, two patients with severe poisoning succumbed despite aggressive interventions, with their deaths attributed to cardiovascular failure secondary to acute hemolysis. Other patients exhibited varying degrees of organ dysfunction, necessitating prolonged hospitalization and intensive care for severely ill individuals. This highlights the severe impact of arsine poisoning and the need for prompt and effective medical responses in such emergencies.

The management of arsine poisoning necessitates prompt and aggressive treatment to mitigate hemolysis and prevent multi-organ failure. In this study, we utilized a combination of TPE, dialysis, and supportive care measures, including transfusions of red blood cells, platelets, and plasma. TPE proved to be effective in removing arsenic from the bloodstream and halting further hemolysis, as demonstrated by reductions in arsenic levels in discarded plasma and the stabilization of hematological parameters [5]. We also noted that severe cases of arsine poisoning presented with elevated WBC and neutrophil counts upon admission, indicating marked immune activation. To address systemic inflammation and hemolysis, all patients received high-dose methylprednisolone. While high-dose corticosteroids are commonly utilized in paraquat poisoning [13], their efficacy specifically in arsine poisoning has not been reported previously and merits further validation through clinical trials. Given that arsine poisoning depletes glutathione stores [4], we incorporated glutathione supplementation along with high-dose vitamin C and vitamin B6 to leverage their antioxidant effects. Previous studies reported relatively high mortality rate of 25%-50% for arsine poisoning cases [3, 5]. Our treatment experience may be helpful for future management of arsine poisoning and for improving patient prognosis.

## Conclusion

This case series describes the clinical manifestations of arsine poisoning in a cluster of 12 patients. The clinical diagnosis was supported by the combination of environmental arsenic detection, clinical hemolysis, and elevated RBC arsenic levels. The comprehensive management included high‑dose methylprednisolone, vitamin C + B6, and chelation therapy for all patients, and additional TPE, dialysis, and other supportive measures when necessary. Given the retrospective design and small sample size, these findings are descriptive. Larger prospective studies are needed to establish standardized treatment protocols.

## Data Availability

The datasets analyzed in the current study are available from the corresponding author on reasonable request.
